# Comparison between endovascular aneurysm repair-selected and endovascular aneurysm repair-only strategies for the management of ruptured abdominal aortic aneurysms: An 11-year experience at a Chinese tertiary hospital

**DOI:** 10.3389/fcvm.2022.870378

**Published:** 2022-08-22

**Authors:** Gang Fang, Jianing Yue, Tao Shuai, Tong Yuan, Bichen Ren, Yuan Fang, Tianyue Pan, Zhenjie Liu, Zhihui Dong, Weiguo Fu

**Affiliations:** ^1^Department of Vascular Surgery, Zhongshan Hospital, Fudan University, Shanghai, China; ^2^Institute of Vascular Surgery, Fudan University, Shanghai, China; ^3^National Clinical Research Center for Interventional Medicine, Shanghai, China; ^4^Department of Vascular Surgery, The Second Affiliated Hospital of Zhejiang University, School of Medicine, Hangzhou, China

**Keywords:** ruptured abdominal aortic aneurysm, ruptured endovascular aneurysm repair, abdominal compartment syndrome, emergent aneurysm repair, stent-graft

## Abstract

**Objectives:**

The aim of this study was to review our management experience of ruptured abdominal aortic aneurysms (RAAAs) using an endovascular aneurysm repair (EVAR)-only strategy, and discuss the feasibility of this strategy.

**Materials and methods:**

A retrospective analysis of clinical data was performed in patients with RAAAs from January 2009 to October 2020. Our strategy toward operative treatment for RAAAs evolved from an EVAR-selected (from January 2009 to April 2014) to an EVAR-only (from May 2014 to October 2020) strategy. Baseline characteristics, thirty-day mortality, perioperative complications, and long-term outcomes of patients were compared between the two periods.

**Results:**

A total of 93 patients undergoing emergent RAAA repair were eventually included. The overall operation rate in RAAAs at our centre was 70.5% (93/132). In the EVAR-only period, all 53 patients underwent ruptured endovascular aneurysm repair (rEVAR). However, only 47.5% (19/40) of patients in the EVAR-selected period underwent rEVAR, and the remaining 21 patients underwent emergent open surgery. Thirty-day mortality in the EVAR-only group was 22.6% (12/53) compared with 25.0% (10/40) for the EVAR-selected group (*P* = 0.79). Systolic blood pressure ≤70 mmHg [adjusted odds ratio (OR) 4.99, 95% confidence interval (CI), 1.13–22.08, *P* = 0.03] and abdominal compartment syndrome (adjusted OR 3.72, 95% CI, 1.12–12.32, *P* = 0.03) were identified as independent risk factors responsible for 30-day mortality. After 5 years, 47.5% (95% CI, 32.0–63.0%) of patients in the EVAR-selected group were still alive versus 49.1% (95% CI, 32.3–65.9%) of patients in the EVAR-only group (*P* = 0.29).

**Conclusion:**

The EVAR-only strategy has allowed rEVAR to be used in nearly all the RAAAs with similar mortality comparing with the EVAR-selected strategy. Due to the avoidance of operative modality selection, the EVAR-only strategy was associated with a more simplified algorithm, less influence on haemodynamics, and a shorter operation and recovery time.

## Introduction

The mortality rates of ruptured abdominal aortic aneurysms (RAAAs) following open surgical repair (OSR) remain high despite advances in surgical and anaesthetic techniques (1). The safety and effectiveness of elective endovascular repair of AAAs have been acknowledged, and ruptured endovascular aneurysm repair (rEVAR) is now considered as an alternative to operative treatment for RAAAs. Meanwhile, the discussion about the role of rEVAR has been sustained during the past two decades due to the discrepancy between randomised controlled trials (RCTs) and real-life studies (2–5). Recently, both the Society for Vascular Surgery (SVS) 2018 practice guidelines and the European Society for Vascular Surgery (ESVS) 2019 clinical practice guidelines on the management of AAAs have strongly recommended (level of recommendation: class 1) rEVAR over OSR for the treatment of RAAAs, when anatomically feasible (6, 7). However, it was reported that less than 50% of RAAA patients were candidates for EVAR based on the neck criteria of instructions for use (IFU) for abdominal aortic endografts (8). Whether the indication of rEVAR could be further extended to a larger group of RAAA patients with more challenging anatomical conditions, remains to be investigated.

Ever since the results of three RCTs (AJAX, ECAR, and IMPROVE) on OSR versus rEVAR appeared in early 2010s (3, 4, 9). A much more aggressive attitude toward rEVAR use has been adopted at our institution. In May 2014, an EVAR-only strategy that involves the complete replacement of OSR for all RAAAs with rEVAR was instituted at our centre, and this strategy is still being used currently. The aim of this study was to compare the outcomes of the “EVAR-only” strategy (from May 2014 to October 2020) with those of the “EVAR-selected” strategy (from January 2009 to April 2014) and discuss the feasibility of the “EVAR-only” strategy.

## Materials and methods

### Study population

A retrospective review of the database of images and electronic medical records was performed to identify patients who received a diagnosis of RAAA between January 2009 and October 2020 at our centre. Clinical data including patient demographics, coexisting medical conditions, patient status upon admission, computed tomography angiography (CTA) images, laboratory test results, surgery details, postoperative complications, and long-term follow-up results were collected. Exclusion criteria included the following: (1) suprarenal RAAAs; (2) ruptured Crawford type I–IV thoracoabdominal aortic aneurysms; (3) symptomatic AAAs without retroperitoneal haematoma or leakage of the contrast agent on preoperative CTA; (4) ruptured isolated iliac artery aneurysms; (5) abdominal aortic pseudoaneurysms without aneurysmal dilatation of the infrarenal abdominal aortic wall; and (6) RAAAs in patients who previously underwent EVAR. Patients meeting any one of these criteria were excluded from the analysis of this study. The rest of the patients receiving a diagnosis of RAAA were then included in the current study. This study was approved by the Committee for the Protection of Human Subjects at Zhongshan Hospital, Fudan University. All patients participating in the study signed an informed consent document.

### Institutional setting

From January 2009 to October 2020, treatment conditions and resources for RAAAs at our centre included the following: (1) a green channel in the emergency room for priority treatment of RAAAs; (2) availability of CTA in the emergency room within 15 min of raising a request; (3) a fully trained on-call multidisciplinary team including vascular surgeons, emergency department physicians, anaesthesiologists, operating room staff, and surgical intensive care unit (sICU) doctors; this team was capable of treating vascular emergencies throughout the day; (4) a fully equipped hybrid operating room used independently by vascular surgeons; (5) priority given for treating RAAAs in the hybrid operating room; (6) at least three brands of endografts with complete specifications stocked in the hybrid operating room; (7) all emergent RAAA repair procedures performed by senior vascular surgeons competent in open and endovascular techniques.

### Treatment strategies

**FIGURE 1:**
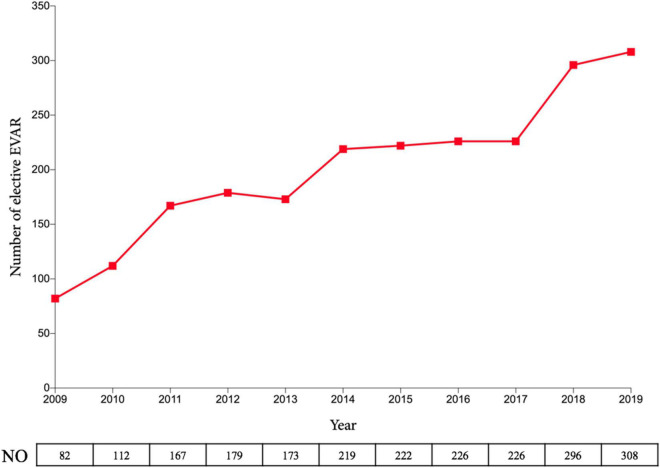
Number of elective EVAR at Zhongshan Hospital over the past decade. EVAR, endovascular aneurysm repair.

From January 2009 to April 2014, we implemented an “EVAR-selected” strategy. The rEVAR procedure was performed in a highly selected patient population during this period, and selection was based on patient’s haemodynamic conditions, neck anatomy, and the senior vascular surgeon’s preference. Haemodynamic instability was defined as a systolic blood pressure ≤70 mmHg for more than 10 min; patients with a systolic blood pressure >70 mmHg were considered haemodynamically stable in the current study. Favourable proximal aneurysm neck anatomy was defined when the well-accepted IFU criteria of endografts were met, which were as follows: (1) proximal neck length ≥10 mm; (2) proximal neck angulation <60°; and (3) proximal neck diameter <32 mm. A hostile proximal neck was defined if any one of the above mentioned criteria were not met. During this period, a rEVAR was performed as the first-choice treatment only in haemodynamically stable patients with a favourable proximal neck. As for haemodynamically unstable patients, the administration of rEVAR was dependent on the personal experience of the senior vascular surgeon. Notably, due to our abundant experience in elective EVAR (Figure 1) for treating AAAs with hostile necks, we tended to prefer rEVAR in haemodynamically stable patients with hostile aneurysm necks to achieve more rapid control of life-threatening haemorrhage.

From May 2014 to September 2020, the strategy for the management of patients with RAAAs could be summarised as “EVAR-only.” Briefly, once the diagnosis of an infrarenal RAAA was established, a rEVAR would be immediately prepared for these patients, regardless of their haemodynamic status and the anatomic conditions of the proximal aneurysm neck. Therefore, rEVAR was the only technique used to repair RAAAs during this period.

### Preoperative assessment

The diagnosis of a RAAA was established when a retroperitoneal haematoma or leakage of the contrast agent was found around a true AAA on CT images. All the patients received CT imaging in the transferring hospital or at our emergency room. An expeditious CTA was mandatory for the haemodynamically stable patients. For the haemodynamically unstable patients, hypotensive haemostasis with strict limitation of fluid infusion was implemented to maintain a target systolic blood pressure of 80–100 mmHg. Surgical interventions including balloon occlusion were performed only when informed consent was obtained from the patients or their family members.

### Emergent repair of ruptured abdominal aortic aneurysms

Emergent open surgery was performed under general anaesthesia. A transperitoneal RAAA repair was utilised in our centre. In haemodynamically unstable patients, transfemoral balloon occlusion of the aorta was considered before anaesthesia induction. Heparin was used selectively according to the patients’ coagulation conditions. When primary abdominal closure was deemed unfeasible due to intestinal oedema, vacuum-pack temporary abdominal wound management with delayed closure was used.

A rEVAR was performed in a hybrid operation room by surgeons with extensive experience in elective EVAR. Transfemoral descending aortic balloon occlusion was performed in haemodynamically unstable patients. The anaesthesia method (local or general) for the following rEVAR procedures was determined by anaesthetists and vascular surgeons, and was mainly based on the degree of dysphoria and haemodynamic conditions in these patients. Technical success was defined as the successful deployment of the endograft into the target segment of the artery without type I and III endoleaks at complete angiogram. Additionally, decompression laparotomy was not performed at the same stage during the rEVAR procedure as during OSR.

### Postoperative management

Patients undergoing rAAA repair were admitted to the sICU. Maintenance of acid-base balance, correction of coagulation function, and improvement of the haemodynamic parameters were the three main goals of our therapy; this aspect has been described in our previous study (10).

Abdominal compartment syndrome (ACS) was defined as an intraluminal bladder pressure value of ≥20 mmHg with a new organ dysfunction (cardiac, respiratory, or renal). For patients with ACS, vascular surgeons and sICU physicians codetermined whether a laparotomy decompression was indicated, mainly according to patients’ tolerance to open surgery, coagulation functions, and willingness of their family members. Patients surviving to discharge after RAAA repair were routinely followed up by CTA at 3, 6, and 12 months, and annually thereafter.

### Statistical analysis

The primary outcome was 30-day all-cause mortality. Secondary outcomes included in-hospital all-cause mortality, postoperative complications during a 30-day postoperative period, and the length of the sICU and hospital stay. Continuous variables were expressed as mean ± SD, and categorical variables were expressed as frequencies and percentages. Categorical variables were compared between groups using the χ^2^ test, and the *t*-test was used to analyse continuous variables. Statistical significance was defined as a *P*-value < 0.05. Univariate and multivariate logistic analyses were used to identify potential independent risk factors of 30-day mortality after emergent repair of RAAAs and the occurrence of ACS after rEVAR. The multivariate logistic model included all variables that exhibited significant differences at the level of *P* < 0.05 in the univariate analysis. Kaplan–Meier analysis was performed to estimate the long-term survival, and the difference between the EVAR-selected and EVAR-only periods was analysed using the log-rank test. Statistical analysis was performed using the SPSS 23 software (IBM Corp., Armonk, NY, United States).

## Results

### Demographic and aneurysmal characteristics

**FIGURE 2:**
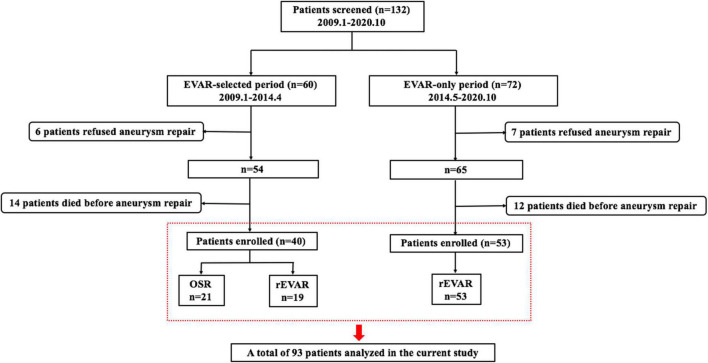
Flowchart of defined groups of patients with ruptured abdominal aortic aneurysms. OSR, open surgical repair; rEVAR, ruptured endovascular aneurysm repair.

A total of 132 patients with RAAAs treated at our institution between January 2009 and October 2020 were identified (Figure 2). Among them, 13 patients refused aneurysm repair and received palliative care, and 26 patients who agreed to surgery died in the emergency room or during transfer into the operating room. After excluding these 39 patients, a total of 93 patients undergoing emergent RAAA repair were eventually included in the current study. Hence, the overall operation rate in RAAAs at our centre was 70.5% (93/132). In the EVAR-only period, all 53 patients underwent rEVAR. However, only 47.5% (19/40) of patients in the EVAR-selected period underwent rEVAR, and the remaining 21 patients underwent emergent open surgery.

**FIGURE 3:**
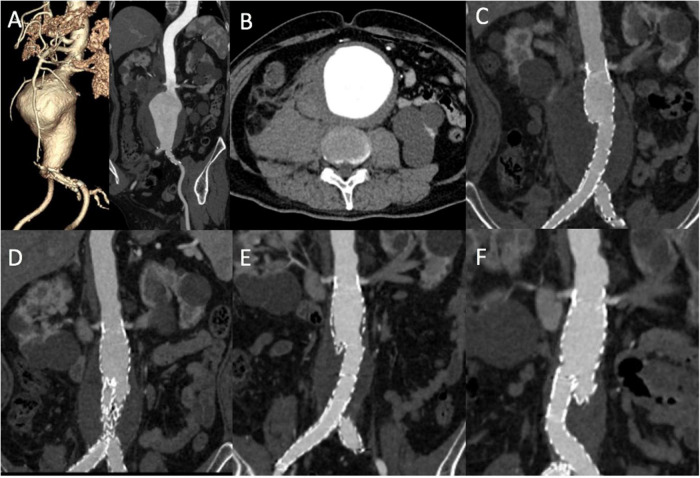
A rEVAR in a patient with a hostile proximal aneurysm neck. **(A,B)** Computed tomography angiography (CTA) showed a ruptured abdominal aneurysm with a short and angulated proximal neck. CTA demonstrated that the size of the aneurysm body with complete thrombosis gradually decreased after rEVAR at 3 months **(C)**, 1 year **(D)**, 2 years **(E)**, and 5 years **(F)**. rEVAR, ruptured endovascular aneurysm repair.

Demographic and aneurysmal characteristics were similar between patients in the EVAR-selected and EVAR-only periods. Hostile proximal necks (Figure 3) were found in approximately 50% of the included patients, which were mostly attributed to neck angulation. The specific information pertaining to this section is displayed in Table 1.

**TABLE 1:** Clinical and aneurysmal characteristics.

	EVAR-selected				EVAR-only (*n* = 53)	*P* ^†^
			
	OSR (*n* = 21)	rEVAR (*n* = 19)	*P* *	Total (*n* = 40)		
Gender						
Male	16 (76.2)	18 (94.7)	0.23	34 (85.0)	46 (86.8)	0.81
Female	5 (23.8)	1 (5.3)		6 (15.0)	7 (13.2)	
Age (years)	64.5 ± 12.0	71.9 ± 9.6	0.04	68.1 ± 11.4	70.4 ± 10.9	0.32
Comorbidities						
Hypertension	21 (100.0)	18 (94.7)	0.23	39 (97.5)	51 (96.2)	1.00
Hyperlipidaemia	13 (62.0)	8 (42.1)	0.10	21 (52.5)	32 (60.4)	0.45
DM	8 (38.1)	5 (26.3)	0.43	13 (32.5)	24 (45.3)	0.21
COPD	0 (0.0)	3 (15.8)	0.10	3 (7.5)	5 (9.4)	1.00
Haemodialysis	1 (4.8)	3 (15.8)	0.53	4 (10.0)	2 (3.8)	0.43
Coronary artery disease	3 (14.3)	2 (10.5)	1.00	5 (12.5)	8 (15.1)	0.72
History of stroke	2 (9.5)	2 (10.5)	1.00	4 (10.0)	5 (9.4)	1.00
Preoperative data						
Hospital transfer	14 (66.7)	14 (73.7)	0.63	28 (70.0)	34 (64.2)	0.55
Time to hospital (hours)	26.1 ± 34.1	27.2 ± 40.4	0.93	26.6 ± 36.7	19.7 ± 21.4	0.26
Time from admission to operation (hours)	1.5 ± 1.1	1.7 ± 1.0	0.91	1.6 ± 1.1	1.3 ± 0.9	0.79
Conscious loss	0 (0.0)	0 (0.0)	1.00	0 (0.0)	8 (15.1)	<0.01
SBP ≤70 mmHg	2 (9.5)	1 (5.3)	1.00	3 (7.5)	10 (18.9)	0.21
Hb ≤90 g/L	5 (23.8)	12 (63.2)	0.01	17 (42.5)	29 (54.7)	0.24
Creatinine >106 mmol/L	13 (61.9)	11 (57.9)	0.80	24 (60.0)	32 (60.4)	0.97
DIC	0 (0.0)	3 (15.8)	0.10	3 (7.5)	1 (1.9)	0.42
Diameter of aneurysms (cm)	7.4 ± 1.8	7.3 ± 2.1	0.81	7.36 ± 1.9	6.9 ± 1.9	0.27
Hostile proximal neck	12 (57.1)	8 (42.1)	0.34	20 (50.0)	27 (50.9)	0.93
Short	6 (28.6)	3 (15.8)	0.56	9 (22.5)	5 (9.4)	0.08
Angulated	9 (42.9)	6 (31.6)	0.46	15 (37.5)	22 (41.5)	0.70
Wide	0 (0.0)	0 (0.0)	1.00	0 (0.0)	1 (1.9)	1.00

Data are presented as n (%) or mean ± SD.

rEVAR, ruptured endovascular aneurysm repair; OSR, open surgical repair; DM, diabetes mellitus; COPD, chronic obstructive pulmonary disease; SBP, systolic blood pressure; Hb, haemoglobin; DIC, disseminated intravascular coagulation.

Chi-square or Fisher’s exact test for categorical variables, and Student’s t-test for continuous variables were used. *P* < 0.05 is considered statistically significant.

P* for OSR versus rEVAR in the EVAR-selected period.

*P*^†^ for EVAR-selected versus EVAR-only.

### Emergent repair

Technical success of the aneurysm repair was achieved in all patients in the EVAR-selected period. Technical failure occurred in four patients (4/53, 7.5%) in the EVAR-only period due to persistent type Ia endoleaks at completion angiogram. Open surgery conversion was not performed in these four patients considering their state of haemorrhagic shock. Patients in the EVAR-only period had significantly less operative time and lower blood transfusion needs (Table 2). When we compared the rEVAR techniques between the two periods (Table 3), a significantly higher incidence of use of local anaesthesia and percutaneous puncture of the femoral artery was observed in the EVAR-only period. Meanwhile, bifurcated endografts were more commonly used in the EVAR-only period compared to the EVAR-selected period, with a statistically significant difference (*P* < 0.01).

**TABLE 2:** Operation outcomes and perioperative complications.

	EVAR-selected				EVAR-only (*n* = 53)	*P* ^†^
			
	OSR (*n* = 21)	rEVAR (*n* = 19)	*P* *	Total (*n* = 40)		
Technical success	21 (100.0)	19 (100.0)	1.00	40 (100.0)	49 (92.5%)	0.50
Operative time	4.9 ± 1.2	2.8 ± 1.0	<0.01	3.9 ± 1.5	2.0 ± 0.8	<0.01
Blood transfusion	1,976.2 ± 1521.8	736.8 ± 1,000.1	<0.01	1,387.5 ± 1,429.0	754.7 ± 1,126.9	0.02
**Perioperative complications**						
ACS	0 (0.0)	7 (36.8)	<0.01	7 (17.5)	18 (34.0)	0.08
Acute kidney injury	4 (19.0)	3 (15.8)	1.00	7 (17.5)	15 (28.3)	0.23
Myocardial infarction	1 (4.8)	0 (0.0)	1.00	1 (2.5)	1 (1.9)	1.00
Stroke	0 (0.0)	0 (0.0)	1.00	0 (0.0)	2 (3.8)	0.50
Respiratory failure	2 (9.5)	4 (21.1)	0.56	6 (15.0)	6 (11.3)	0.60
Acute lower limb ischaemia	2 (9.5)	0 (0.0)	0.49	2 (5.0)	0 (0.0)	0.18
Wound infection	3 (14.3)	0 (0.0)	0.23	3 (7.5)	0 (0.0)	0.08
Transient paraplegia	1 (4.8)	0 (0.0)	1.00	1 (2.5)	0 (0.0)	0.43
Gastrointestinal haemorrhage	2 (9.5)	1 (5.3)	1.00	3 (7.5)	2 (3.8)	0.65
Length of sICU stay	12.9 ± 20.5	4.3 ± 4.7	0.07	8.8 ± 15.7	7.3 ± 11.7	0.61
Length of hospital stay	22.1 ± 22.2	12.6 ± 9.3	0.09	17.6 ± 17.7	12.2 ± 15.5	0.13
In-hospital mortality	6 (28.6)	5 (26.3)	0.87	11 (27.5)	15 (28.3)	0.93
30-day mortality	5 (23.8)	5 (26.3)	0.86	10 (25.0)	12 (22.6)	0.79

Data are presented as n (%) or mean ± SD.

rEVAR, ruptured endovascular aneurysm repair; OSR, open surgical repair; ACS, abdominal compartment syndrome; sICU, surgical intensive care unit.

Chi-square or Fisher’s exact test for categorical variables, and Student’s t-test for continuous variables were used. *P* < 0.05 is considered statistically significant.

P* for OSR versus rEVAR in the EVAR-selected period.

*P*^†^ for EVAR-selected versus EVAR-only.

**TABLE 3:** Characteristics of rEVAR.

	EVAR-selected	EVAR-only	*P*
	(*n* = 19)	(*n* = 53)	
Anaesthesia			
Local	0 (0.0)	21 (39.6)	<0.01
General	19 (100.0)	32 (60.4)	
Access type			
Percutaneous	3 (15.8)	46 (86.8)	<0.01
Cut-down	16 (84.2)	7 (13.2)	
Suprarenal balloon occlusion	2 (10.5)	13 (24.5)	0.34
Stent-graft configuration			
Bifurcated	10 (52.6)	46 (86.8)	<0.01
Tube	3 (15.8)	5 (9.4)	
Aorto-uni-iliac	6 (31.6)	2 (3.8)	
Renal artery coverage	0 (0.0)	2 (3.8)	1

Data are presented as n (%).

EVAR, endovascular aneurysm repair.

Chi-square or Fisher’s exact test for categorical variables was used. *P* < 0.05 is considered statistically significant.

### Early mortality

Thirty-day mortality in the EVAR-only period was 22.6% (12/53) compared with 25.0% (10/40) in the EVAR-selected period (*P* = 0.79). There was also no significant difference (*P* = 0.93) in in-hospital mortality between the EVAR-only (15/53, 28.3%) and EVAR-selected (11/40, 27.5%) periods (Table 2).

Univariate logistic regression analysis showed loss of consciousness (Glasgow Coma Scale score ≤8) at admission, SBP ≤90 mmHg, serum creatinine >186 mmol/L, Hb ≤60 g/L, and ACS were risk factors related to 30-day mortality (Table 4). Notably, the EVAR or the EVAR-only strategy was not identified as a risk factor of 30-day mortality after univariate analysis. Multivariate logistic regression analysis revealed that unstable haemodynamics [adjusted odds ratio (OR) 4.99, 95% confidence interval (CI), 1.13–22.08, *P* = 0.03] and ACS (adjusted OR 3.72, 95% CI, 1.12–12.32, *P* = 0.03) were independent risk factors of 30-day mortality in the current cohort of patients.

**TABLE 4:** Logistic regression of the 30-day mortality.

Characteristics	Unadjusted OR	95% CI	*P*	Adjusted OR	95% CI	*P*
Female	1.43	0.40–5.16	0.59			
**Age (years)**						
≤70	Reference					
70–80	1.68	0.57–5.00	0.35			
>80	3.14	0.84–11.72	0.09			
Coronary artery disease	2.15	0.63–7.40	0.22			
Haemodialysis	0.59	0.07–5.34	0.64			
COPD	1.02	0.19–5.42	0.99			
Stroke	2.74	0.67–11.22	0.16			
Conscious loss	6.20	1.35–28.45	0.02	2.94	0.42–20.71	0.28
**SBP (mmHg)**						
>90	Reference			Reference		
70–90	5.04	1.58–16.10	<0.01	2.72	0.70–10.47	0.15
≤70	8.50	2.21–32.67	<0.01	4.99	1.13–22.08	0.03
**Creatinine (mmol/L)**						
≤106	Reference			Reference		
106–186	4.00	1.10–14.49	0.04	1.53	0.31–7.61	0.60
>186	4.00	1.08–14.85	0.04	3.30	0.74–14.84	0.12
**Hb (g/L)**						
>90	Reference			Reference		
60–90	2.12	0.72–6.25	0.18	1.03	0.28–3.86	0.96
≤60	11.43	2.30–56.70	<0.01	2.74	0.40–18.99	0.31
**Aneurysm diameter (cm)**						
≤6.5	Reference					
6.5–8.5	2.53	0.83–7.67	0.10			
>8.5	1.04	0.26–4.19	0.96			
Hostile proximal neck	1.74	0.67–4.56	0.26			
General anaesthesia	0.94	0.30–2.92	0.91			
ACS	6.28	2.24–17.64	<0.01	3.72	1.12–12.32	0.03
EVAR	1.07	0.34–3.33	0.91			
EVAR-only strategy	0.33	0.38–2.52	0.96			

OR, odds ratio; CI, confidence interval; COPD, chronic obstructive pulmonary disease; SBP, systolic blood pressure; Hb, haemoglobin; DIC, disseminated intravascular coagulation; ACS, abdominal compartment syndrome; EVAR, endovascular aneurysm repair.

*P* < 0.05 is considered statistically significant.

### Perioperative complications

The specific data pertaining to complications are displayed in Table 2. In general, there was no significant difference in the rates of recorded postoperative complications between the two periods. ACS was the most common postoperative complication (25/93, 26.9%), and all instances of ACS occurred in the rEVAR group. Advanced age (>75 years), Hb level <90 g/L upon admission, massive blood transfusion (>1,500 ml), and intraoperative balloon occlusion were significantly associated with postoperative ACS. When adjusted for covariates, age >75 years (adjusted OR 4.13, 95% CI, 1.23–13.91, *P* = 0.02) and Hb <90 g/L (adjusted OR 6.49, 95% CI, 1.65–25.51, *P* < 0.01) were identified as independent risk predictors of ACS (Table 5).

**TABLE 5:** Logistic regression of ACS in the rEVAR group.

Characteristics	Unadjusted OR	95% CI	*P*	Adjusted OR	95% CI	*P*
Female	2.05	0.47–9.00	0.34			
Age >75 years	6.20	2.14–17.99	<0.01	4.13	1.23–13.91	0.02
Conscious loss	3.67	0.80–16.86	0.10			
SBP ≤70 mmHg	2.65	0.72–9.78	0.14			
Creatinine >186 mmol/L	2.29	0.82–6.40	0.11			
Hb <90 g/L	7.09	2.10–23.90	<0.01	6.49	1.65–25.51	<0.01
Aneurysm diameter >6.5 cm	2.56	0.87–7.56	0.09			
Hostile neck	2.62	0.96–7.14	0.06			
General anaesthesia	2.18	0.77–6.23	0.14			
AUI	0.59	0.11–3.19	0.54			
Access type	0.10	0.35–2.82	0.99			
Blood transfusion >1,500 ml	4.73	1.37–16.53	0.01	2.66	0.61–11.72	0.20
Balloon occlusion	5.60	1.65–19.06	<0.01	2.61	0.62–11.06	0.19
EVAR-only strategy	0.88	0.30–2.63	0.82			

ACS, abdominal compartment syndrome; rEVAR, ruptured endovascular aneurysm repair; OR, odds ratio; CI, confidence interval; SBP, systolic blood pressure; Hb, haemoglobin; AUI, aorto-uni-iliac.

*P* < 0.05 is considered statistically significant.

Additionally, despite the fact that no significant difference was observed in the length of the sICU and hospital stay between the two periods, the mean length of hospital stay was about five days shorter in the EVAR-only period.

### Long-term survival

**FIGURE 4:**
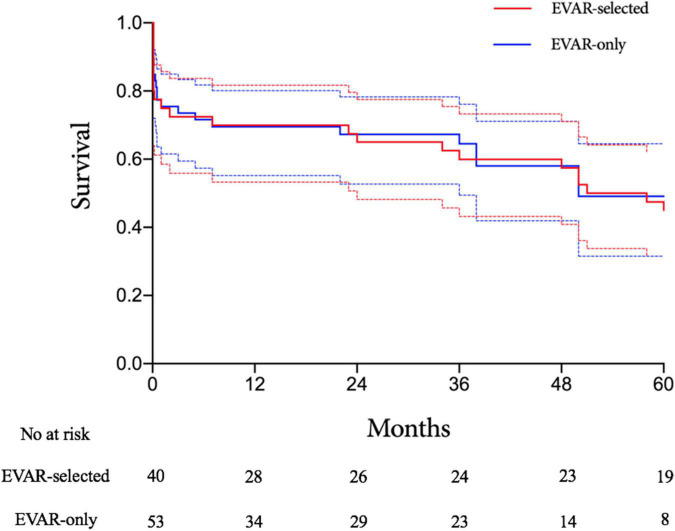
Kaplan–Meier analysis of long-term survival rates for the EVAR-selected versus EVAR-only groups. EVAR, endovascular aneurysm repair.

There were 29 and 38 patients surviving to discharge in the EVAR-selected and EVAR-only periods, respectively. The median follow-up time was 61 months (range, 7–121 months) in the EVAR-selected period and 39 months (range, 2–77 months) in the EVAR-only period. The Kaplan–Meier analysis showed no significant difference in mid-term survival between the two periods (log-rank *P* = 0.88, Figure 4). The estimated 3-year survival rates were 60% (95% CI, 44.9–75.1%) in the EVAR-selected group compared with 64.5% (95% CI, 51.2–77.8%)in the EVAR-only group (*P* = 0.55). After 5 years, 47.5% (95% CI, 32.0–63.0%) of patients in the EVAR-selected period were still alive versus 49.1% (95% CI, 32.3–65.9%) of patients in the EVAR-only period (*P* = 0.29).

## Discussion

**FIGURE 5:**
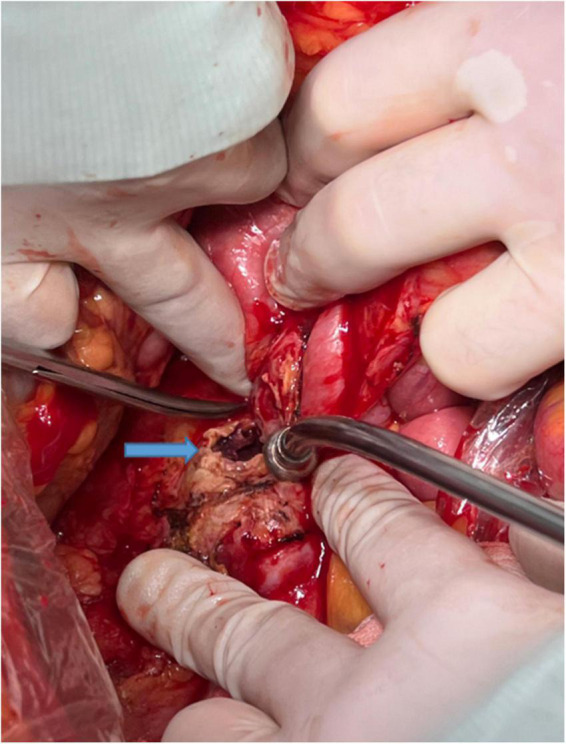
The rupture site (blue arrow) of the arterial wall in a ruptured abdominal aortic aneurysm.

Although no early mortality benefits over OSR (Figure 5) have been demonstrated in RCTs, rEVAR has been increasingly used for the treatment of RAAAs in recent years due to its minimally invasive nature and requirement of a shorter hospital stay. In most institutions, RAAA patients with specific characteristics such as stable haemodynamics and anatomically feasible aneurysm necks have been selected for rEVAR. Hence, the strategy for the management of RAAAs has gradually evolved from the OSR-only to EVAR-selected strategies after the introduction of rEVAR. Implementation of a well-designed EVAR-selected strategy incorporating rEVAR with OSR has been reported to significantly improve perioperative morbidity and mortality. It was reported that the 30-day mortality ranged from 14.3 to 35.3% after implementation of a structured EVAR-selected strategy for the management of RAAAs (11–14). In the current study, the 30-day mortality in the EVAR-selected period was 25%, which was consistent with previous studies.

An EVAR-only strategy for RAAAs was first reported by Mayer et al. (12). Non-selected RAAAs were treated with rEVAR for over a period of 32 months at two centres, and favourable outcomes were achieved. The 30-day mortality in the EVAR-only period was comparable with that in the EVAR/OPEN period (24.3 versus 25.5%, *P* = 0.83), which, to some extent, implied that the superior efficacy of rEVAR for RAAAs in historical reports might be attributed to this new technique rather than to patient selection. However, the implementation of this radical EVAR-only approach for RAAAs has not been reported at other centres. In the current study, the 30-day mortality in the EVAR-only period was lower than that in the EVAR-selected period, although the difference was not statistically significant. Additionally, similar long-term survival rates were observed between the two periods, which was not reported in the previous study by Meyer et al. (12).

It was estimated that more than half of the RAAAs were not candidates for EVAR with current endograft devices, due to unfavourable anatomy (15). An increasing number of RAAAs without IFU for endografts are being treated with rEVAR at other experienced vascular centres, as at our centre. Perrott et al. reported a reduction in 30-day mortality for EVAR-suitable patients following OSR compared with EVAR-unsuitable patients, although the difference was not statistically different (6.9 versus 30.4%, *P* = 0.07) (16). However, it was also reported that the 30-day mortality in patients with a friendly anatomy after OSR was comparable with those with a hostile anatomy (30 versus 38%, *P* = 0.23) (17). As for patients treated with rEVAR, Broos et al. observed similar 30-day mortality between the favourable and hostile neck groups (14 versus 12%, *P* = 1.00) (18). However, Baderkhan et al. did not find a significant difference in 30-day mortality between patients inside (15%) and outside (30%) the IFU (*P* = 0.09), and the 3-year mortality was observed to be significantly lower in EVAR-suitable patients (33.8 versus 56.0%, *P* = 0.02); an aneurysm neck diameter >29 mm was found to be an independent risk factor of overall mortality. In the IMPROVE trial (19), no association was found between five morphological parameters (maximum aortic diameter, aneurysm neck diameter, conicality, proximal neck angle, and maximum common iliac diameter) and mortality. Only a shorter proximal neck length was identified as an independent risk factor for overall 30-day mortality. In addition, for RAAAs treated with rEVAR, the 30-day mortality was found to be higher if the neck length decreased by less than 10 mm. The threshold was 15 mm for RAAAs treated with OSR. Based on the IMPROVE data, it seemed that a shorter neck length had an adverse impact on postoperative survival, regardless of the operative modality. In the current study, a hostile proximal neck was not identified as a risk factor of 30-day mortality. However, it was noted that all instances of technical failure occurred in patients with a hostile proximal neck, which suggested that hostile anatomy might negatively affect the technical success rates of rEVAR. Findings from our analysis demonstrated that unstable haemodynamics and postoperative ACS, but not the implementation of rEVAR or the EVAR-only strategy, were associated with significantly increased 30-day mortality. Meanwhile, the independent risk factors of ACS following rEVAR were advanced age (>75 years) and moderate or severe anaemia (Hb <90 g/L). We believe that with reference to the prognosis of RAAAs following operation, the effects of particular preoperative clinical characteristics have far outweighed those of the type of operative modality. The performance of rEVAR should not be restricted by the emergent state of patients at major centres that have abundant elective endovascular experience.

Abdominal compartment syndrome was a common complication in patients with RAAAs. rEVAR was associated with a higher incidence of postoperative ACS compared with OSR due to the untreated retroperitoneal haematoma. A meta-analysis of ACS after rEVAR by Karkos et al. estimated that the ACS rate might be higher than 20% with improved awareness and vigilant monitoring (20). In the current study, ACS was observed by routine monitoring of intraluminal bladder pressure in about 35% of patients treated with rEVAR, which was consistent with the study by Karkos et al. (20). The optimal management strategy for ACS remains debatable. Although decompression laparotomy has been recommended by some investigators for patients with sustained high bladder pressure (12, 21), this recommendation and treatment decision should be carefully evaluated considering that decompressive bleeding might be more uncontrollable in RAAA patients, and may lead to high mortality.

The current study has several limitations, including its retrospective design which could have biassed patient selection. Second, despite the relatively large sample size of the present study, it remains too limited to generate more convincing statistical results. Third, our study extended over 11 years. There may have been learning curves for using some techniques during this period, as reflected by the fact that a significantly higher proportion of rEVAR cases in the later period were performed using local anaesthesia, percutaneous access, and bifurcated endografts. Additionally, endograft evolution during the past decade might have had a sizeable influence on the favourable outcomes in the EVAR-only period (22). However, it is difficult to further analyse the influence of these factors in this retrospective study.

## Conclusion

The EVAR-only strategy has allowed rEVAR to be used in nearly all the RAAAs with similar mortality comparing with the EVAR-selected strategy. Due to the avoidance of operative modality selection, the EVAR-only strategy was associated with a more simplified algorithm, less influence on haemodynamics, and a shorter operation and recovery time. Prospective validation of the EVAR-only strategy is required at more experienced vascular centres.

## Data availability statement

The original contributions presented in the study are included in the article/supplementary material, further inquiries can be directed to the corresponding authors.

## Ethics statement

This study design was approved by the Ethics Committee for the Protection of Human Subjects at Zhongshan Hospital, Fudan University, Shanghai, China. All included patients were informed about the nature of the study and gave their written informed consent.

## Author contributions

GF, JY, TS, TY, BR, YF, TP, and ZL collected the data. GF and JY wrote the manuscript. GF, JY, ZD, and WF critically reviewed the manuscript and contributed significantly to discussion. All authors contributed to the article and approved the submitted version.
